# Everolimus and Sunitinib potentially work as therapeutic drugs for infantile hemangiomas

**DOI:** 10.1038/s41390-025-04028-7

**Published:** 2025-04-05

**Authors:** Rongfang Xie, Zhujue Taohuang, Rosalind Kieran, Zhiyu Li, Luying Wang, Changxian Dong, Jianfeng Ge, Xusheng Wang, Miaomiao Li

**Affiliations:** 1 School of Pharmaceutical Sciences (Shenzhen), Shenzhen Campus of Sun Yat-sen University, Sun Yat-sen University, Shenzhen, Guangdong 518000 China; 2 Early Cancer Institute, Department of Oncology, University of Cambridge, Cambridge, UK; 3 Cambridge University Hospitals NHS Foundation Trust, Cambridge, UK; 4 Department of Burn and Plastic Surgery, Shandong Provincial Hospital, Shandong University, Jinan, Shandong 250021 China; 5 Department of Hemangioma and Vascular Malformation Surgery, People’s Hospital of Zhengzhou University, Zhengzhou University, Zhengzhou, Henan 450000 China

## Abstract

**Background:**

Infantile hemangiomas (IH) are common vascular tumors in infants, with no well-defined therapeutic agents currently available. Recent studies have explored molecular mechanisms involved in IH progression, but the lack of immortalized hemangioma-derived endothelial cell (iHemEC) models has limited drug discovery efforts.

**Methods:**

We established an immortalized hemangioma-derived endothelial cell (iHemEC) expressing hemangioma markers and screened 18 potential drugs. Transcriptome profiling and Gene Set Enrichment Analysis (GSEA) were applied to assess the molecular effects of Everolimus and Sunitinib.

**Results:**

Sunitinib, Elimusertib, HIF-1 inhibitor-4, Rebastinib, and Everolimus inhibited iHemEC with lower IC_50_ than Propranolol and Rapamycin. GSEA showed that PI3K/AKT/mTOR pathway was only downregulated in Everolimus treated cells. Chromosome instability was found specifically in Sunitinib treated cells, which has been reported to cause DNA damage. DNA damage induced ROS and extracellular ROS production was only observed in Sunitinib treated cells. Additionally, Sunitinib can trigger P53 activation and BCL2 downregulation with a dose of 0.2 µM which is fifty times lower than the dose of Everolimus at 10 µM.

**Conclusion:**

We successfully developed an iHemEC model for in vitro drug screening and mechanistic study. Everolimus and Sunitinib emerged as promising therapeutic candidates for IH, providing a valuable basis for future research.

**Clinical perspectives:**

Infantile hemangiomas (IH) are very common tumors in the neonatal period, with an incidence of approximately 2% to 10% among newborns, there are no well-defined therapeutic agents for IH, nor are there established human immortalized cell lines for in vitro studies. We establish an immortalized hemangioma-derived endothelial cell (iHemEC) which highly express markers of hemangioma. Drug screening was performed on iHemEC, Everolimus and Sunitinib were found efficiently induce cell death to iHemEC with much lower IC_50_ than front line drug Propranolol. Bulk RNAseq and WB analysis showed that Everolimus specifically inhibit PI3K/AKT/mTOR pathway, however Sunitinib induce chromosome instability and DNA damage. Both drugs can trigger P53 dependent cell death. Our study successfully developed an iHemEC cell line suitable for in vitro drug screening and mechanistic study. Sunitinib, VEGFR inhibitor, potentially can applied for the treatment of IH.

**Impact:**

- Developed a novel immortalized hemangioma-derived endothelial cell (iHemEC) model that replicates key IH features, overcoming limitations of primary cell models.
- Identified Sunitinib and Everolimus as promising therapeutic candidates with superior efficacy, supported by transcriptome and protein analyses.
- Revealed distinct drug mechanisms, with Everolimus targeting PI3K/AKT/mTOR and Sunitinib inducing chromosome instability and DNA damage.

## Introduction

Infantile hemangiomas (IH) are common tumors in the neonatal period, characterized by benign proliferation of capillary endothelial cells and their supporting tissues, with an incidence of approximately 2 to 10% among newborns.^1^ Typically, IH are not visible at birth but emerge in early infancy (2–7 weeks old) and proliferate from 4 to 18 months of age, reaching about 80% of their final size before gradually involuting over the next 3 to 9 years.^2–4^ However, post-involution, about 10 to 15% of untreated children may experience residual skin and subcutaneous tissue changes, such as obstruction, ulceration, or disfigurement.^3,4^ The pathogenesis of IH shares similarities with malignant tumors, particularly in angiogenesis and inflammation. Overexpression of vascular endothelial growth factor (VEGF) in proliferating IH lesions promotes abnormal vasculogenesis, while dysregulation of inflammatory pathways, particularly through NFkB, plays a role in both tumor progression and vascular anomalies. These molecular parallels suggest VEGF and NFkB as potential therapeutic targets.^5^

Clinically, various pharmacological treatments have been utilized for IH, including propranolol, vincristine, and interferon.^6–8^ Propranolol, a non-selective beta-blocker, is widely regarded as the first-line treatment for IH. Its potential mechanisms include vasoconstriction, inhibition of angiogenesis through suppression of vascular endothelial growth factor (VEGF) and basic fibroblast growth factor (bFGF), and induction of apoptosis of endothelial cells and hemangioma-derived stem cells.^9^ Despite its widespread use, propranolol is still not suitable for all IH patients.^10^ Vincristine and interferon, though effective, are typically reserved for severe cases due to their associated toxicity and side effects.^7,11^

Most studies on IH are based on the isolation of tumor cells from infants. However, primary IH cell lines can only be efficiently expanded to around passage 6-8 which prevents researchers from performing systematically in vitro studies. Immortalized cell models can be employed in CRISPR-cas9 based genome-wide screening to search for potential therapeutic targets and can aid in evaluating the efficacy and safety of drug candidates, providing potential avenues for discovering new treatments for IH. Moreover, they can serve as disease models, assisting researchers in better understanding and simulating the onset and progression of the disease. This is of significance for studying the pathogenesis and developing therapeutic strategies for IH.

To address this issue, in this study, primary HemEC cells were infected with a lentiviral cassette containing SV40 large T antigen and hTERT. We successfully selected a stable, immortalized HemEC cell line (iHemEC) that maintained high expression levels of GLUT-1, CD31, and VEGFR2,^12^ providing a reliable cell model for IH research. Using the established cell line, we screened 18 small molecules and identified compounds more potent than Rapamycin in inhibiting cell proliferation, including Sunitinib, Elimusertib, HIF-1 inhibitor-4, Rebastinib and Everolimus. The mechanisms of action of Everolimus and Sunitinib validated for inflammatory modulation of iHemEC, providing insights into the disease progression and potential clinical treatments for IH. In summary, this study offers a stable cell model for fundamental research on IH and introduces new possibilities for developing clinical treatment strategies. The stable and reproducible nature of iHemEC makes them ideal for high-throughput genetic screening, potentially leading to the identification of critical genes and pathways that drive IH development and progression.

## Materials and methods

### Clinical samples and cell lines

Specimens of IH were obtained under a protocol approved by the Committee on Clinical Investigation at Henan Provincial People’s Hospital, China. The clinical diagnosis was confirmed by H&E staining and GLUT1 immunohistochemistry in the Department of Pathology. Informed consent was obtained for use of IH specimens. HemEC were isolated from IH specimens as previously described and all cells were tested negative for mycoplasma. Briefly, IH specimens were minced and digested overnight with 5 mg/mL dispase II solution(04942078001, Roche). Approximately 1mm^3^ pieces were further minced using surgical scissors and subjected to enzymatic digestion with 1 mg/mL collagenase I for 2 h at 37 °C (SCR103, Sigma). The harvest cells were cultured in Endothelial Cell Medium (1001, ScienCell) supplemented with foetal bovine serum (FBS), ECGS and penicillin-streptomycin according to the manufacturer’s recommendations. Cells positive for VEGFR2, CD31, and GLUT-1, as detected by western blot and immunofluorescence staining, were defined as HemEC.

Human HEK293T (CRL-11268) and HUVEC (PCS-100-013) were cultured in Dulbecco’s Modified Eagle Medium (DMEM; 11965118, Gibco) supplemented with 10% fetal bovine serum (FBS; C04001-500, Viva Cell) and 1% penicillin–streptomycin at 37°C with 5% CO_2_. Jurkat (Clone E6-1, TIB-152) cell lines were grown in RPMI 1640 medium (11875119, Gibco) at the same conditions. All these cell lines were purchased from American Type Culture Collection (ATCC, Rockville, MD).

### Antigen infection with lentivirus

SV40 Large-T and hTERT antigens used in this study were obtained from Ubigene Biosciences Co., Ltd., Guangzhou, China. Lentiviral preparation and amplification were performed in HEK293 T cells using the Lipofectamine 2000 kit (11668019, Invitrogen). The media was collected 72 h post-transfection, followed by centrifugation at 500 g for 10 min to isolate the virus-containing supernatant, which was then filtered through a 0.45 μm filter. The virus was stored at −80 °C until needed. When primary HemEC were cultured to 70% confluency, virus was added for infection, and the medium was replaced with fresh medium after 12 h. 72 h post-infection, infected cells were selected using G418 (GC26403, GLPBIO).

### The detection of cellular-growth curves for distinct cell lines

2 × 10^3^ cells were seeded per well in a 96-well plate. The media containing 0.5 mg/mL MTT was replaced every 24 h. After a 4 h incubation period, 150 μL of Dimethyl sulfoxide (DMSO) was added to each well to dissolve the formazan crystals. The absorbance of the resulting solution was measured at 570 nm using a microplate reader (BioTek Epoch 2). Each experiment was performed in triplicate.

### Cytotoxicity assay

A dose-dependent cytotoxicity was examined using the MTT assay. iHemEC were placed at a density of 6 × 10^3^ cells per well in 96-well plates. After 24 h of culture, cells were treated with various concentrations of the test compounds for 72 h, as listed in Supplementary Table S1. Absorbance of the resulting formazan was measured at 570 nm using Microplate reader. IC_50_ values were calculated using Graphad 10.1.2 statistical software. Each experiment was performed in triplicate.

### RNA-seq analysis

The final concentrations of Everolimus and Sunitinib chosen for the treatment of iHemEC for transcriptome profiling were 10 µM, with an intervention time of 24 h, which was determined based on the results of the above inhibition assay. After treatment, the cells were washed with PBS and collected using 1 mL of TRIzol Reagent (15596018CN, Invitrogen) to extract total RNA according to the manufacturer’s instructions. RNA quality was assessed on an Agilent 2100 Bioanalyzer (Agilent Technologies, Palo Alto, CA) and checked using RNase free agarose gel electrophoresis. Then the mRNA enriched by Oligo (dT) was fragmented into short fragments using fragmentation buffer and reversely transcribed into cDNA by using NEBNext Ultra RNA Library Prep Kit for Illumina (E7530, New England Biolabs). The purified double-stranded cDNA fragments were end repaired, had a base added, and were ligated to Illumina sequencing adapters. The ligation reaction was purified with the AMPure XP Beads (A63880, Beckman). And polymerase chain reaction (PCR) amplified. The resulting cDNA library was sequenced using Illumina Novaseq6000 (Repugene Technology, Hangzhou, China).

### Differential gene expression analysis

As previously reported,^13,14^ edgeR was used to perform differential gene expression analysis between samples of different TOAST classes. Using an exact test that is comparable to Fisher’s exact test, we estimated common dispersion for every gene in edgeR and tested for differential expression on every transcript. Genes with a count > 0 across all examined samples were taken into consideration for each pairwise comparison. Genes with a false discovery rate (using the Benjamini-Hochberg method) corrected *p* value (*q*) < 0.05 and an absolute fold-change in expression ≥ 1.5 were considered differentially expressed genes (DEGs).

### Bioinformatics analyses

Gene Ontology (GO) is an international framework for the functional classification of genes, which explain the attributes of genes and their products in any organism. GO has three ontologies: molecular function, cellular component and biological process. Each GO-term is a part of a specific ontology type.

Typically, genes work in concert with one another to perform certain biological tasks. Kyoto Encyclopedia of Genes and Genomes (KEGG) is the major public pathway-related database. Analysis based on pathways contributes to our understanding of the biological roles of genes. When compared to the total genome background, pathway enrichment analysis revealed substantially enhanced metabolic or signal transduction pathways in DEGs.

R package clusterProfiler (version 3.14.3)^15^ was used to do GO and KEGG enrichment analysis. Statistical significance was defined as a *p*-value of less than 0.05 and a false discovery rate (FDR) of less than 0.25.

### Dihydrorhodamine 123 staining for reactive oxygen species (ROS) detection

Dihydrorhodamine 123 (DHR 123, GC30581, GLPBIO) was used for the detection of ROS according to the manufacturer’s protocol. Briefly, primary HemEC were seeded on a 35 mm glass-bottom dishes. After 24 h of treatment with Sunitinib and Everolimus (10, 20, 40 μM), the samples were washed with PBS. Images were obtained using a confocal laser scanning microscope (Zeiss, LSM880).

### Colony formation

iHemEC were seeded at a density of 500 cells per well in 6-well plates and evenly dispersed by gently shaking. After 2-week incubation period, the cells were fixed with 4% paraformaldehyde for 15 min and subsequently stained with Crystal Violet Staining Solution for another 15 min. Lastly, the dye was then washed off with PBS, and colonies were counted using a standard optical microscope.

### Western blotting analysis

The iHemEC were seeded at a density of 2.0 × 10^5^ cells per well in 6-well plates. Upon reaching 80% confluency, the cells were treated with Sunitinib (0.2, 1, 10, 20 μM), Everolimus (0.2, 1, 10, 20 μM) and Rapamycin (20 μM). After 24 h, the cells were lysed with ice-cold RIPA buffer containing a protease and phosphatase inhibitor cocktail (P002, NCM). Standard techniques were used for western blot analysis. The primary and secondary antibodies used are listed in Supplementary Table S2. The signals were assessed using the WesternBright ECL (Advansta, K-12045-D50) and normalized to β-actin or GADPH.

### Flow cytometry

Proportion of cell death after drug action analyzed using an apoptosis and necrosis detection kit (C1056, Beyotime). Primary HemEC treated with Sunitinib (10, 40, 80 μM) and Everolimus (10, 40, 80 μM) for 24 h were digested with trypsin, centrifuged at 300 g for 5 min at 4 °C, and washed twice with cold PBS. Subsequently, the cells were resuspended in1×binding buffer. Next, 5 µL of Hoechst and 5 µL of PI staining solution were added and incubated with resuspended cells at room temperature for 10–15 min. The prepared samples were analyzed within 1 h using a BD FACSAri Fusion flow cytometer (BD Biosciences).

### Immunofluorescence staining

Primary HemEC were seeded in 35 mm glass-bottom dishes and cultured for 2 days until reaching confluency. Following treatment, the cells were fixed in 4% paraformaldehyde for 15 min. The cells were then permeabilized and blocked for 1 h at 37°C using 0.5% Triton X-100 in PBS (pH 7.4) supplemented with 10% normal goat serum. Subsequently, the cells were incubated overnight at 4 °C with primary antibodies: VEGFR2 (9698, 1:200, CST), CD31 (sc-53411, 1:50, Santa Cruz), Ki67 (9129, 1:100, CST) and GLUT-1 (MAB-0813, 1:50, MXB). After three washes with PBS, the specimens were exposed to Alexa Fluor 488 Anti-mouse IgG (ab150113, 1: 500, Abcam) and Alexa Fluor 647 Anti-rabbit IgG (ab150075, 1:500, Abcam) in PBS containing 3% bovine serum albumin for 1 h at 37 °C. Cell nuclei were stained with DAPI (G1012, Servicebio). Images were acquired using an Olympus FV3000 confocal microscope. Negative controls were prepared using the same immunostaining protocol, omitting the primary antibodies.

### Statistical analysis

All data were analyzed using GraphPad Prism (version 10.1.2; GraphPad Software). One-way analysis of variance (ANOVA) was employed for statistical comparisons of the groups. Statistical significance was set at a *p*-value (*<0.05, **<0.01, ***<0.001).

## Results

### HemEC isolation and characterization

**Fig. 1:**
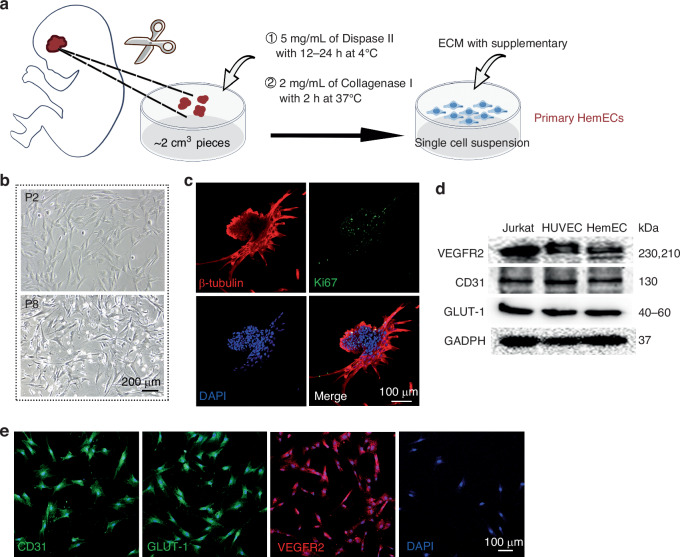
Isolation and characterization of primary HemEC. **a** Schematic diagram illustrating the process for isolating primary HemEC. **b** Bright-field images depicting primary HemEC at passages 2 (P2) and passages 8 (P8). Scale bar: 200 μm. **c** Observation of spontaneous clumping in primary HemEC. Scale bar: 100 μm. **d** Western blot analysis demonstrating the expression of multiple HemEC-specific markers. **e** Immunofluorescence staining confirming the presence of HemEC-specific markers. Scale bar: 100 μm.

Figure 1a illustrates the techniques for isolating HemEC from IH. As shown in Fig. 1b, the isolated primary HemEC exhibited the typical endothelial cell morphology, characterized by elongated and slender spindle shapes. However, around the eighth passage, they began to exhibit signs of reduced growth and apoptosis. Figure 1c shows that the primary HemEC appear to cluster spontaneously and have some potential for angiogenesis when they are cultivated at a density of more than 90% for longer than three days. These cells were identified as HemEC using immunofluorescence and western blot since they displayed high levels of key proteins as GLUT-1, VEGFR2, and CD31 (Fig. 1d and 1e).

### Generation of iHemEC

**Fig. 2:**
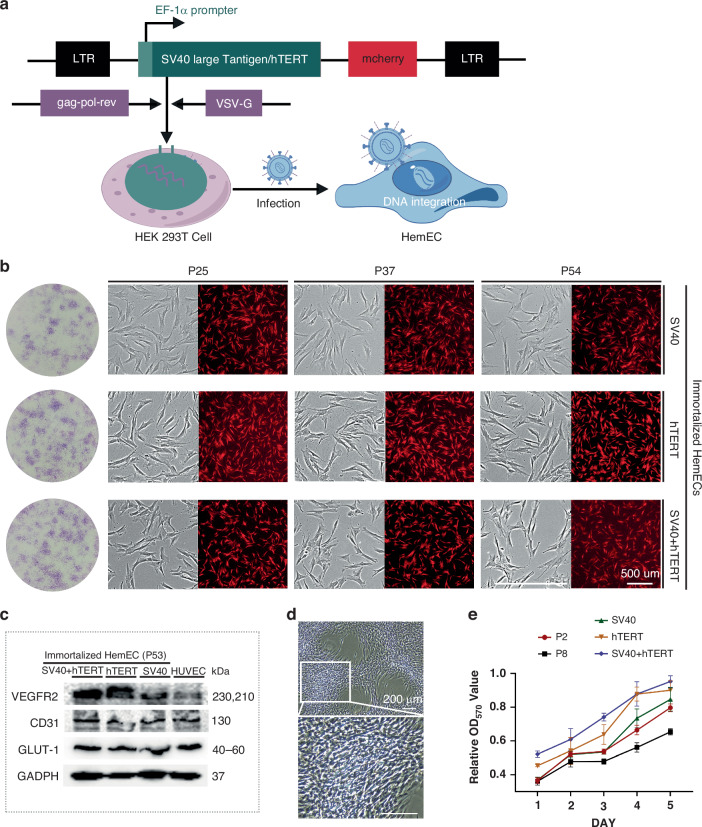
Establishment and characterization of iHemEC. **a** Immortalization strategies for primary HemEC, including the construction of hTERT plasmid and SV40 large T antigen plasmid (SV40), followed by lentiviral packaging and infection of primary HemEC. **b** Proliferation characteristics of iHemEC obtained *via* different immortalization methods. Colony formation assay demonstrating the proliferative capacity of iHemEC. Comparative analysis of iHemEC at different passages using both fluorescence and bright-field microscopy. Scale bar: 500 μm. **c** Western blot analysis showing the expression of HemEC marker proteins in iHemEC. **d** Spontaneous aggregation of iHemEC (SV40+hTERT) forming vessel-like structures. The image shows phase-contrast microscopy of iHemEC cells exhibiting spontaneous clustering and alignment, resembling vessel-like structures. The inset highlights a magnified view of the cellular arrangement, demonstrating dense aggregation and elongated morphology. Scale bar: 200 μm. **e** Growth curves comparing primary HemEC with iHemEC.

Primary HemEC were single or double transduced by SV40 or hTERT lentivirus in order to create a stable and sustainable cell model (Fig. 2a). Neomycin analogue G418 was used to select the cells after they displayed mCherry red fluorescence as a result of transduction. The selected cell line retained the traits of primary HemEC, including morphological features (Fig. 2b), marker protein expression (CD31, VEGFR2, and GLUT-1) (Fig. 2c), and angiogenic potenial (Fig. 2d). Crucially, steady passaging to over 50 generations and a strong proliferative potential were displayed by the iHemEC (Fig. 2b), suggesting that the production of immortalized cells was accomplished. In stark contrast to primary cells (P8), immortalized cells created using both SV40 and hTERT viruses exhibited the quickest proliferation within 5 days when compared to other methods (Fig. 2e). Thus, iHemEC will be a valuable in vitro model system in which to search drug candidates and study pathomechanisms of the IH.

### Screening for drugs with inhibitory effects based on iHemEC

**Fig. 3:**
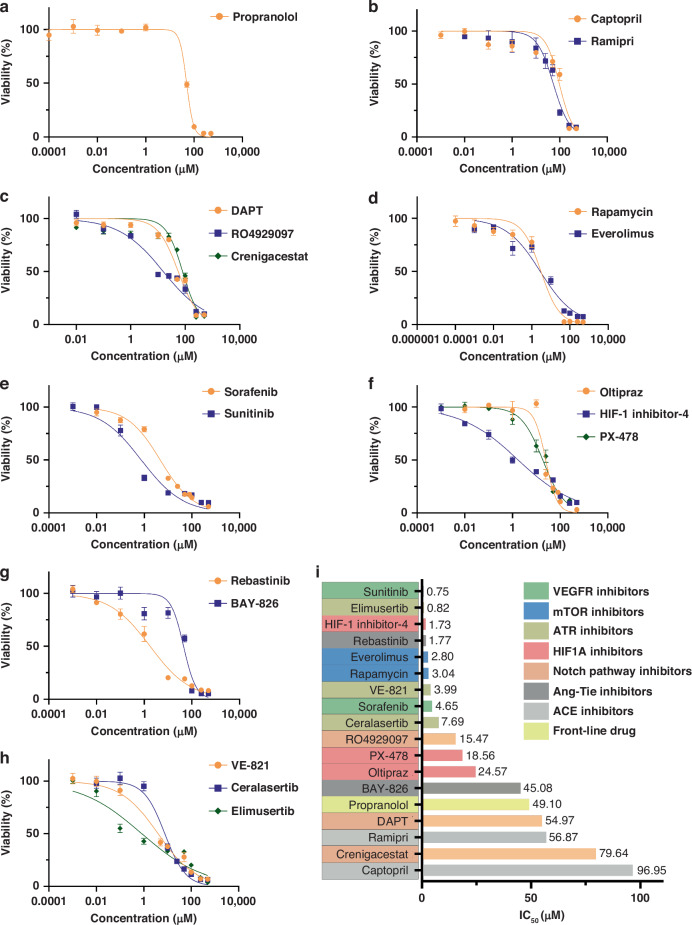
Drug candidates screening for IH. **a** IC_50_ Determination of Propranolol (β-receptor antagonists). **b** IC_50_ Determination of Captopril and Ramipril (ACE inhibitors). **c** IC_50_ Determination of DAPT, RO4929097 and Crenigacestat (Notch signaling pathway inhibitors). **d** IC_50_ Determination of Rapamycin and Everolimus (mTOR signaling pathway inhibitors). **e** IC_50_ Determination of Sorafenib and Sunitinib (VEGF signaling pathway inhibitors). **f** IC_50_ Determination of Oltipraze, HIF-1 inhibitor-4 and PX-478 (HIF signaling pathway inhibitors). **g** IC_50_ Determination of Rebastinib and BAY-826 (Tie-2 kinase inhibitors). **h** IC_50_ Determination of VE-821, Ceralasertib and Elimusertib (ATR inhibitors). **i** Summary bar graph showing the IC_50_ values for each compound derived from the dose-response curves in panels **a–h**. The IC_50_ values provide a comparative measure of the potency of each drug in reducing iHemEC viability after 72 h of exposure. Data from three independent experiments are represented as mean ± SD.

Increased proliferation capability of iHemEC makes drugs screening possible. We have chosen 18 drugs candidates which target HIF, VEGF, Notch, mTOR, Tie-2, ACE and ATR which are potential vulnerable pathways, which iHemEC might have according to previous studies.^16–20^ MTT assay was conducted to evaluate the IC_50_ values of various drugs. Propranolol, the first-line treatment for IH, is also a β-receptor antagonists, with an IC_50_ value of 49.1 μM on iHemEC (Fig. 3a). ACE inhibitors and Notch inhibitors showed similar IC_50_ value (Fig. 3b, c and i). Rapamycin, an mTOR inhibitor, has been used to treat vascular tumors and vascular malformations in recent years^21^ and has an IC_50_ value of 3.04 μM (Fig. 3d). This implies that Rapamycin might be a better control than Propranolol for iHemEC screening of medication candidates. Drugs with a lower IC_50_ than Rapamycin are Sunitinib (0.75 μM), Elimusertib (0.82 μM), HIF-1 inhibitor-4 (1.73 μM), Rebastinib (1.77 μM), Everolimus (2.80 μM), and therefore could be taken into consideration as candidates for iHemEC inhibition. (Fig. 3d, h). The IC_50_ values of all 18 agents on iHemEC are visualized in Fig. 3i. It also suggests that ATR inhibitors, VEGF inhibitors, and mTOR inhibitors, all of which have low IC_50_ values, suggest that ATR-Mediated DNA Damage Response, HIF signaling pathway, the VEGF signaling pathway, and the PI3K/AKT/mTOR signaling pathway, might be the key pathways to treat IH.

### Transcriptomic analysis on Everolimus and Sunitinib treated iHemEC

**Fig. 4:**
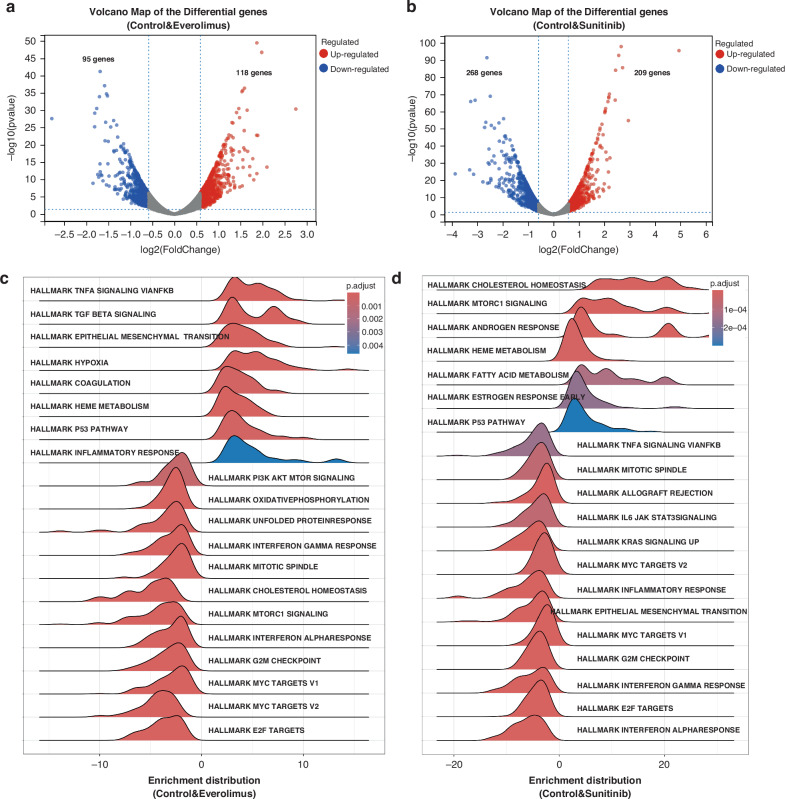
Transcriptomic analysis of Everolimus and Sunitinib treated iHemEC. **a** Volcano plots of DEGs in Everolimus versus Control. Genes with upregulated expression are on the right side of the dashed line, and genes with downregulated expression are on the left side. **b** Volcano plots of DEGs in Sunitinib versus Control. Genes with upregulated expression are on the right side of the dashed line, and genes with downregulated expression are on the left side. **c** Ridgeline plot for KEGG analysis in Everolimus versus Control. Pathways enriched with upregulated genes are shown on the right side of the plot, while pathways enriched with downregulated genes are shown on the left side. **d** Ridgeline plot for KEGG analysis in Everolimus versus Control. Pathways enriched with upregulated genes are shown on the right side of the plot, while pathways enriched with downregulated genes are shown on the left side.

To understand the working mechanism of the tested drugs, Sunitinib and Everolimus treated samples were chosen for bulk RNAseq analysis. These two drugs have IC_50_ values of 2.8 μM and 0.75 μM respectively which are much lower than IC_50_ value of Rapamycin. Differential gene expression analysis show that 118 and 95 genes are significantly upregulated and downregulated respectively in Everolimus treated cells (Fig. 4a). There are even more differentially expressed genes in Sunitinib treated cells, of which there are 209 upregulated and 268 downregulated genes (Fig. 4b).

GSEA analysis using MsigDB shows that HALLMARK of MYC TARGET V1 and V2, G2M CHECKPOINT, E2F TARGETS are significantly downregulated in both Everolimus and Suntinib treated cells which indicates cell cycle inhibition. HALLMARK of P53 pathway is significantly activated in two both drugs which indicates that both drugs’ mechanism of cytotoxicity might rely on P53 pathway activation (Fig. 4c and d).

We also performed GSEA_KEGG and GSEA_GO pathway analysis, KEGG of DNA replication, Citrate cycle TCA cycle, Cell cycle et al. and GO_BP of Mitochondrial gene expression and translation, and Ribosome biogenesis are significantly suppressed in Everolimus treated cells (Supplemental Fig. S1a and S1b) which suggest that Everolimus can inhibit iHemEC cell cycle, ATP production and gene transcription et al. Sunitinib also inhibits Cell cycle and DNA replication in our GSEA_KEGG analysis (Supplemental Fig. S2a). Interestingly, Chromosome separation, Chromosome segregation, Regulation of chromosome segregation, Chromosome region, Sister chromatid segregation et al. are significantly suppressed in our GO analysis which suggests that Sunitinib can inhibit cell proliferation *via* negatively affecting chromosome instability (Supplemental Fig. S2b).

### Everolimus suppresses PI3K-AKT-MTOR pathway on iHemEC

**Fig. 5:**
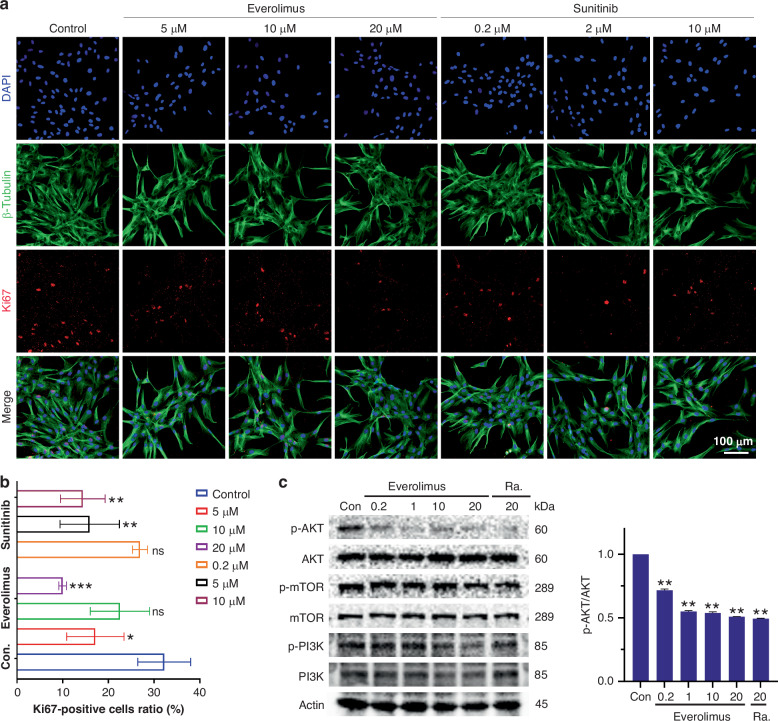
Everolimus and Sunitinib inhibit proliferation in HemEC. **a** Ki67 staining under after Everolimus (5, 10, 20 μM) and Sunitinb (0.2, 2, 10 μM) treatment for 24 h. Scale bar: 100 μm. **b** Ratio quantification of Ki67-positive cells after Everolimus and Sunitinib treatment. Data from three independent experiments are represented as mean ± SD. The average and standard deviation from at least three repeats are shown in the data, and standard one-way ANOVA is used to evaluate the differences. *p < 0.05, **p < 0.01, ***p < 0.001. **c** Western blotting analysis of p-AKT, AKT, p-mTOR, mTOR, p-PI3K, PI3K and Actin in HemEC treated with 0.2, 1,10, 20 μM Everolimus, and 20 μM Rapamycin for 24 h. The right panels display the quantitative analysis of the p-AKT/AKT ratio. Data are expressed as mean ± SD (*n* = 3). Statistical analysis was performed using one-way ANOVA. *p < 0.05, **p < 0.01, ns indicates no significant difference.

From the transcriptomic level, we know that Everolimus and Sunitinib can inhibit the cell cycle of iHemEC. To validate if both drugs can inhibit iHemEC proliferation at the protein level, we performed Ki67 immunofluorescence staining on both drugs treated cells. Corresponding to MYC downregulation, the ratio of Ki67-positive cells was significantly reduced after both Everolimus and Sunitinib interventions (Fig. 5a and b).

Everolimus is an mTOR inhibitor, and correspondingly, the HALLMARK PI3K AKT MTOR SIGNALING pathway was significantly downregulated in Everolimus treated cells (Supplemental Fig. S3a), but not in Sunitinib. PI3K-AKT-MTOR is crucial for the growth, multiplication, survival, and metabolism of tumor cells. To validate the inactivation of the HALLMARK PI3K AKT MTOR SIGNALING pathway, we analysed the phosphorylation of AKT, mTOR and PI3K by western blot, all three p-AKT, p-mTOR, p-PI3K were downregulated in a dose-dependent manner when cells treated with Everolimus, particularly p-AKT can be detected reduction at 0.2 μM (Fig. 5c and d) which is comparable with the Rapamycin inhibition at 20 μM. However, it was high concentrations of Sunitinb (20 μM) that reduced p-AKT expression (Supplemental Fig. S3b) which fits with our bulk RNAseq pathway analysis. Potentially, Everolimus inhibits iHemEC proliferation mainly *via* suppression of the PI3K-AKT-MTOR pathway.

### Sunitinib triggers apoptotic activation on iHemEC at a much lower dose than Everolimus

**Fig. 6:**
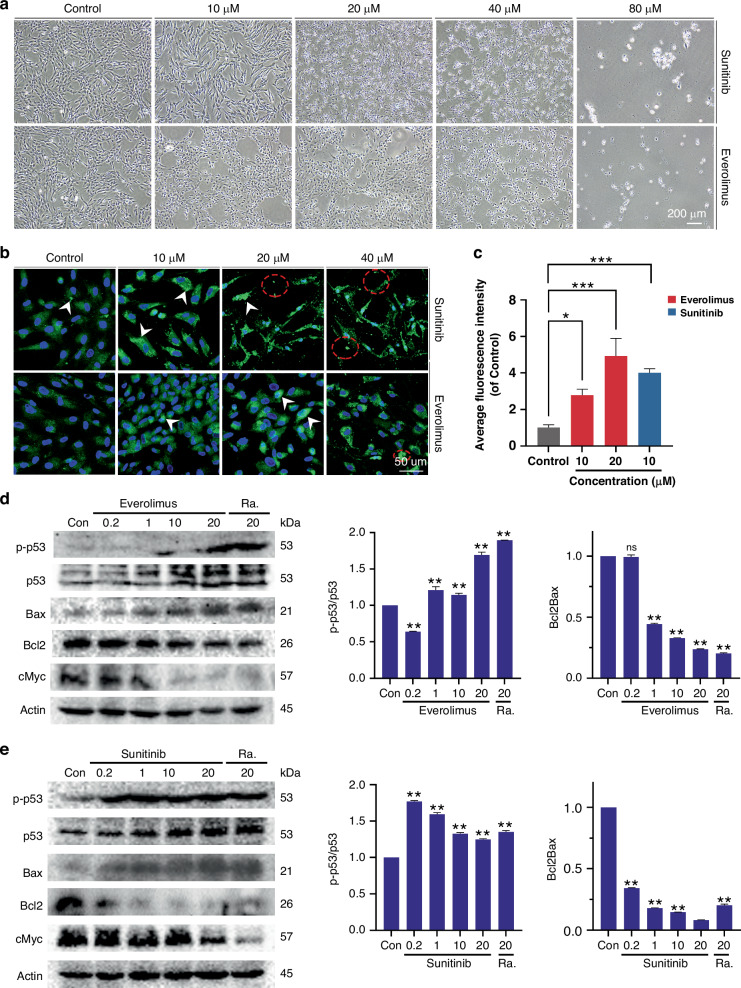
Sunitinib and Everolimus promote apoptosis in HemEC. **a** Bright-field microscopy images illustrating the dose-dependent effects of Sunitinib and Everolimus on cell morphology at concentrations of 10 µM, 20 µM, 40 µM and 80 µM. Scale bar: 200 μm. **b** DHR123 detects reactive oxygen species in HemEC after Everolimus and Sunitinib treatment (10, 20, 40 μM). White arrows point to ROS, red dashed circles refer to extracellular ROS. Scale bar: 50 μm. **c** Quantification of average fluorescence intensity corresponding to ROS levels in cells treated with varying concentrations of Sunitinib and Everolimus, relative to control. The average and standard deviation from at least three repeats are shown in the data, and standard one-way ANOVA is used to evaluate the differences.*p < 0.05, **p < 0.01, ***p < 0.001. **d** Western blot analysis of p53 signaling pathway activation in response to 24-h Everolimus treatment at concentrations of 0.2 µM, 1 µM, 10 µM, and 20 µM. Levels of phosphorylated p53 (p-p53), total p53, pro-apoptotic Bax, anti-apoptotic Bcl2, and cMyc are shown, with actin serving as a loading control. Rapamycin (Ra) at 20 µM is included as a positive control for mTOR inhibition. **e** Western blot analysis of p53 signaling pathway activation in response to 24-h Sunitinib treatment at concentrations of 0.2 µM, 1 µM, 10 µM, and 20 µM. The expression levels of p-p53, total p53, Bax, Bcl2, and cMyc are presented, with actin used as a loading control. Rapamycin (Ra) at 20 µM is included for positive control. The right panels display the quantitative analysis of the p-p53/p53 ratio and the Bcl2/Bax ratio. Data are expressed as mean ± SD (*n* = 3). Statistical analysis was performed using one-way ANOVA. *p < 0.05, **p < 0.01, ns indicates no significant difference.

iHemEC underwent morphological change as a result of Everolimus and Sunitinib as compared to the untreated group. These changes included a tendency for iHemEC to become elliptical rather than spindle-shaped, in addition to a gradual crumpling with increasing concentration that ultimately resembled necrosis and apoptosis (Fig. 6a). When compared to Everolimus at the same dose, Sunitinib clearly resulted in more pronounced alterations to cell shape, which at 20 μM ultimately led to cell death (Fig. 6a and Supplemental Fig. S4a and S4b). Compared to Everolimus group, the fraction of dead cells in Sunitinib group was significantly higher and displayed a dose dependent inhibition (Supplemental Fig. S4a and S4b).

Interestingly, chromosome instability was only specifically enriched in Sunitinib treated samples (Supplemental Fig. S2) and it was reported to lead to DNA damage,^22^ and DNA damage can induce ROS.^23^ DHR123 staining was used to measure the ROS level in our study. Sunitinib and Everolimus can both raise the levels of ROS in HemEC. The HemEC cell membrane ruptured under the action of Sunitinib (20 μM) and Everolimus (40 μM), the contents flowed out, and the fluorescence produced by ROS was lost. As a result, the fluorescence intensity of Sunitinib (10 μM) and Everolimus (10 μM and 20 μM) was counted. Statistical analysis also showed that Sunitinib produced stronger fluorescence than Everolimus at the same concentration of 10 μM action (Fig. 6b and c), which indicates that Sunitinib might induce more DNA damage due to chromosome instability.

P53 plays an essential role for intrinsic pathway of apoptosis which is significant enriched in GSEA Hallmark analysis on both Everolimus and Sunitinib treated iHemEC (Fig. 4c and d). It is the direct marker of cell death. Western blot shows that total P53 and p-P53, the active form of P53, are upregulated in both drug treatments at 10 μM (Fig. 6d and e). Furthermore, the pro-apoptotic protein, BAX, is clearly upregulated in Sunitinib treated samples (Fig. 6e). Correspondingly to P53 activation, BCL2, the anti-apoptotic protein, is reduced in both drugs, but the reduction is dramatically stronger in Sunitinib treated cells. Furthermore, it is quite noticeable around 0.2 μM which suggests the higher vulnerability of iHemEC to VEGFR inhibition. The MYC gene has a significant carcinogenic function in a range of malignancies which also has a dose dependent expression in response to the two drugs.

## Discussion

IH occurs in approximately 2 to 10% newborns. However, there are still no well-defined drugs for IH and there are also no established human immortalized cell lines for in vitro studies. In our study, we successfully created an iHemEC line by expressing SV40 and hTERT cassette. The iHemEC can be split for over 50 passages while still retaining high expression levels of markers such as VEGFR2, CD31, and GLUT-1 which make this cell line a good model of drugs screening.

It has been reported that IH could be vulnerable to inhibition of HIF1A pathway, VEGFR, Notch signaling, mTOR pathway, Angiopoietin-tie pathway, ACE pathway and ATR.^16–20^ We screened 18 drugs candidates which cover all these pathways. Interestingly, propranolol, which is a first-line drug for IH, can only inhibit iHemEC IC_50_ at around 49.1 μM and therefore compared to the other 17 drugs candidates, Propranolol seems to be not one of the best options. If we define any drug which inhibits the growth of iHemEC as having an IC_50_ of less than 10 μM as a ‘working’ candidate, then we find that both VEGFR inhibitors (2/2), both mTOR inhibitors (2/2) and both ATR inhibitors (2/2) inhibit iHemEC efficiently, as they all have an IC_50_ of less than 10 μM. On the other hand, all of the Notch pathway inhibitors (0/3) and both ACE inhibitors (0/2) can only inhibit iHemEC IC_50_ with high concentrations. Only one HIF1A inhibitor (1/3) and only one Ang-Tie pathway inhibitor (1/3) can inhibit iHemEC. Therefore, we believe VEGFR, mTOR and ATR pathway can be vulnerable targets in iHemEC (Fig. 3i and Supplemental Fig. S4c). For subsequent validation experiments, a drug concentration of 10 µM was used for a 24 h. This concentration was chosen to ensure robust activation or inhibition of key intracellular signaling pathways, as lower concentrations often result in insufficient or inconsistent responses, making quantification challenging.

We chose Everolimus and Sunitinib, the mTOR and VEGFR inhibitor respectively, for bulk RNAseq analysis. Everolimus might inhibit iHemEC through PI3K Akt mTOR pathway because HALLMARK PI3K AKT MTOR SIGNALING was significantly downregulated in Everolimus treated cells (Supplemental Fig. S3a). This pathway was also validated by western blot. ATR inhibitors were found to be ‘working drugs’ in our screening. ATR inhibitors can prevent DNA damage repair and cause genomic stability which indicates that iHemEC is vulnerable to DNA damage response. Interestingly, chromosome instability and DNA damage induced ROS was specifically found in in Sunitinib treated cells (Supplemental Fig. S2b) which again reflects the importance of DNA damage response in iHemEC apoptosis. P53 dependent apoptosis was validated in both drugs treated cells through western blot (Fig. 6d and e). Except for the potential DNA damage induced apoptosis by Sunitinib, some other pathways like PPAR signaling, IL-17 signaling et al. could be also the cause of iHemEC apoptosis (Supplemental Fig. S4d). It is still not clear whether other pathways play an essential role for the survival of iHemEC. In future studies, we aim to further validate and explore the underlying mechanisms using a CRISPR/Cas9-based genome-wide screening to identify key pathways involved in the therapeutic response of iHemEC. This approach provides a more systematic and comprehensive exploration of critical signaling pathways, complementing the findings from the Western blot analysis.

In conclusion, we established iHemEC that facilitates drug screening and mechanistic studies of IH. Everolimus and Sunitinib were identified as potential therapeutic drugs that suppress iHemEC through PI3K Akt mTOR inhibition and DNA damage induction and then lead to P53 dependent apoptosis. This work opens up new avenues for developing therapeutic treatment plans and provides a stable cell model for basic research on IH. Further research is essential to optimize these treatments for clinical application, ensuring their safety and efficacy for pediatric use.

## Supplementary information

Supplemental Figures

Supplementary tables

## Data Availability

All materials, data, and protocols are present in the manuscript or available upon request. The RNA-seq data generated within this project have been uploaded into the Gene Expression Omnibus under the access Code GSE279596.
