# Supplementary material for: Everolimus and Sunitinib potentially work as therapeutic drugs for infantile hemangiomas
Source: Pediatr Res. 2025 Apr 5;98(6):2374–84. doi: 10.1038/s41390-025-04028-7 (PMC12811115; doi:10.1038/s41390-025-04028-7)
Supplement: Supplementary file 1 — Supplemental Figures [file 41390_2025_4028_MOESM1_ESM.pdf]

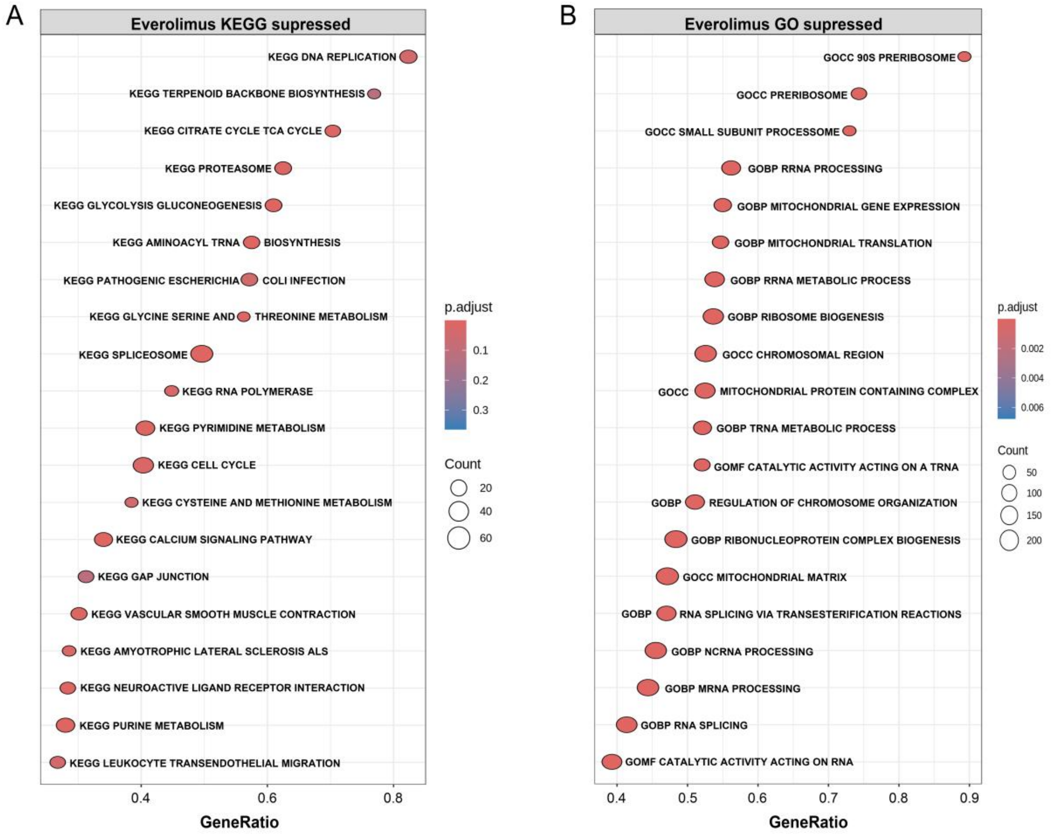

Figure S1

GSEA of KEGG pathways and GO suppression by Everolimus treatment. **(A)** Dot plot representing the suppression of KEGG pathways in response to Everolimus treatment. The pathways are ranked according to the GeneRatio, indicating the proportion of genes involved in each pathway relative to the total number of genes associated with that pathway. **(B)** Dot plot showing the suppression of GO biological processes by Everolimus treatment. The processes are ordered by GeneRatio. The color gradient reflects the padj, where darker red indicates higher statistical significance. Dot size correlates with the count of genes within each pathway.

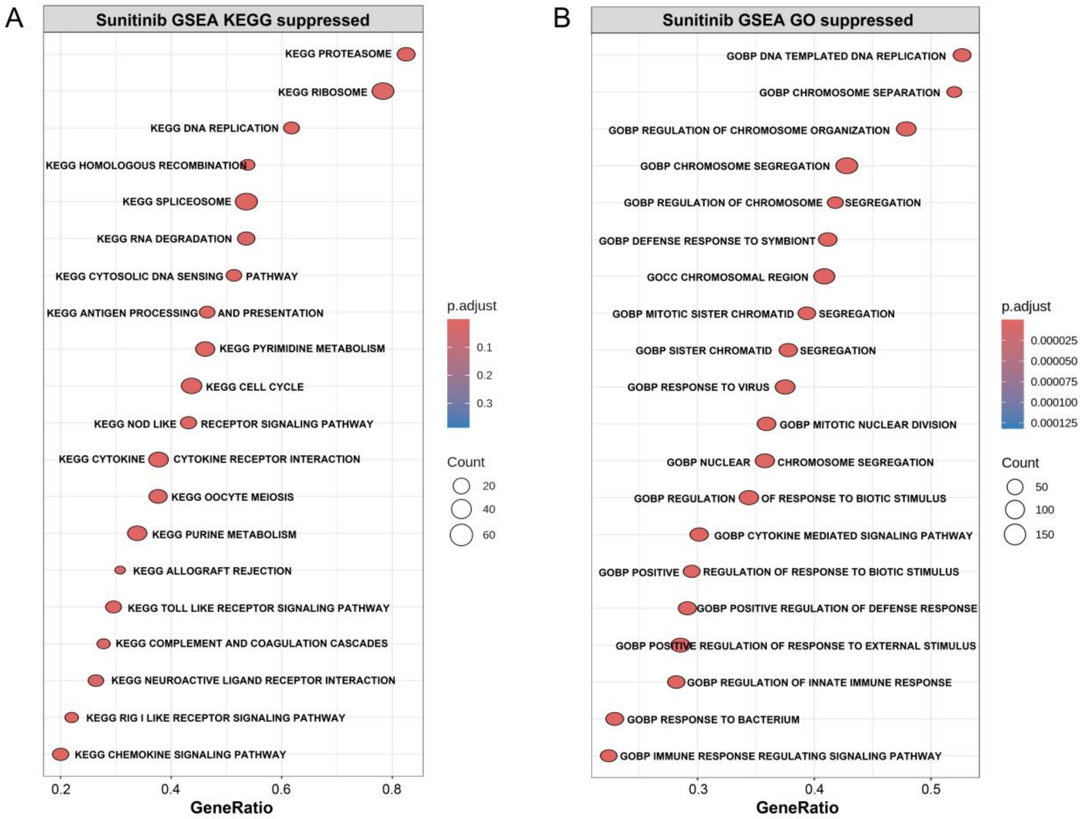

Figure S2

GSEA of KEGG pathways and GO biological process suppression by Sunitinib treatment. **(A)** Dot plot representing the suppression of KEGG pathways in response to Everolimus treatment. **(B)** Dot plot showing the suppression of GO biological processes by Everolimus treatment. The color gradient reflects the padj, where darker red indicates higher statistical significance. Dot size correlates with the count of genes within each pathway.

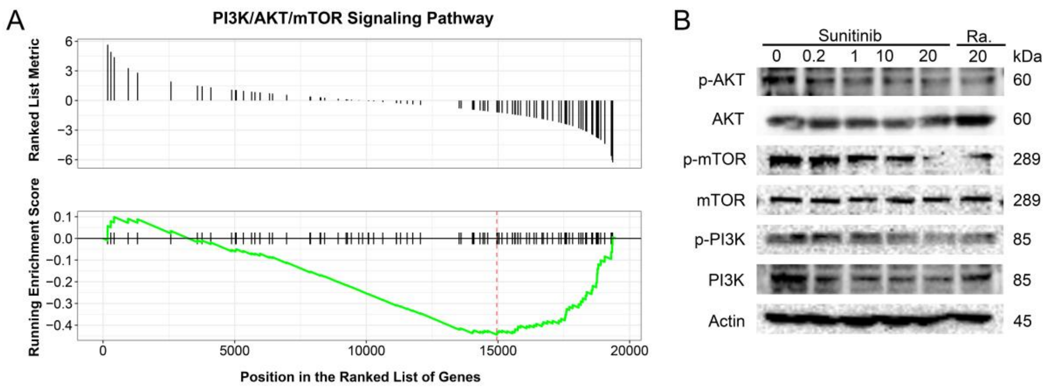

Figure S3

Impact of Everolimus on PI3K/AKT/mTOR Signaling Pathway. **(A)** GSEA plot for the hallmark gene set PI3K/AKT/mTOR following Sunitinib treatment. The upper panel displays the ranked list metric, showing the distribution of genes across the ranked list. The lower panel depicts the running enrichment score (green curve), highlighting the enrichment of PI3K/AKT/mTOR pathway genes at the lower end of the ranked gene list. The position of the peak in the enrichment score curve indicates the point of maximum enrichment for this pathway. **(B)** Western blot analysis showing the effects of Sunitinib on key proteins in the PI3K/AKT/mTOR signaling pathway. The phosphorylation levels of AKT (p-AKT), mTOR (p-mTOR), and PI3K (p-PI3K) are presented relative to their total protein levels. Actin serves as a loading control. The doses of Sunitinib are indicated, with an additional comparison to Rapamycin treatment as a control.

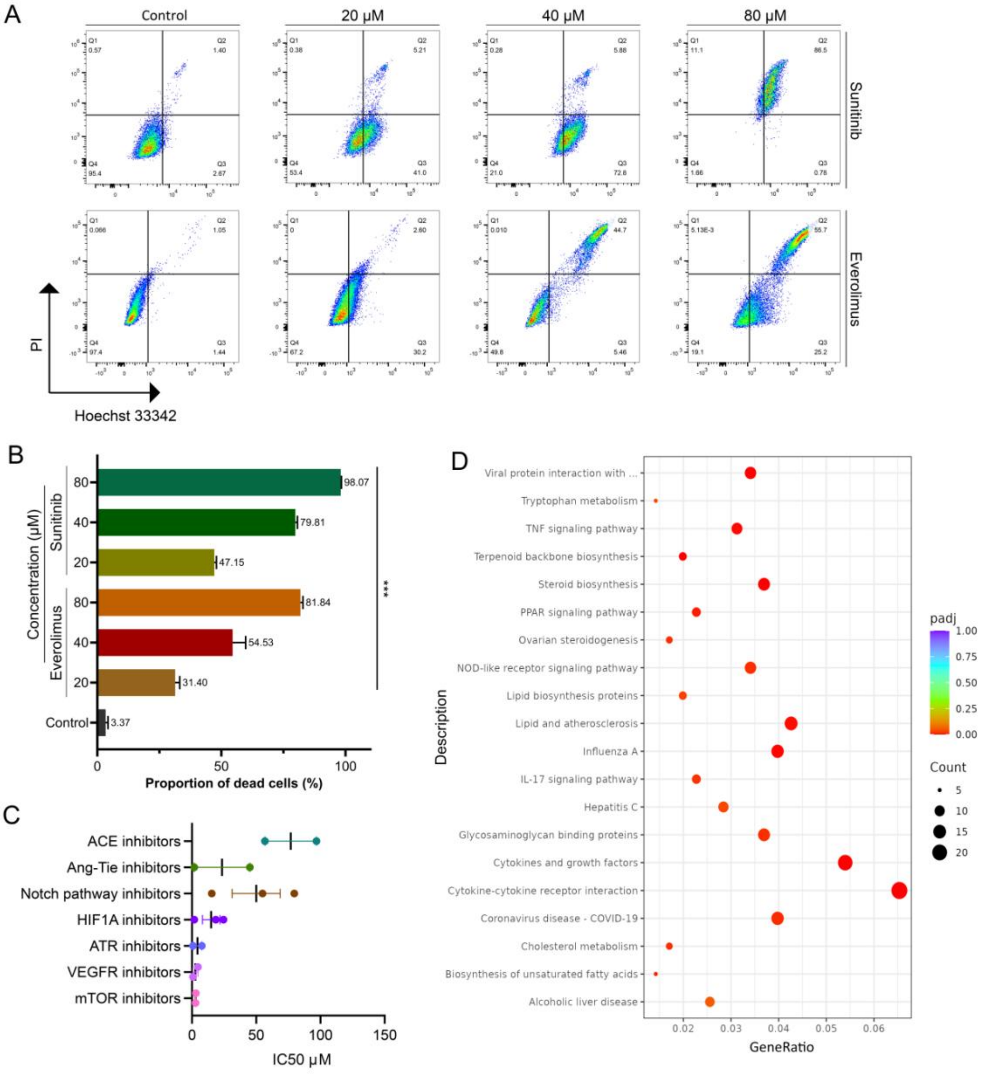

Figure S4

Cell death analysis and pathway enrichment following 24 h treatment with Sunitinib and Everolimus. **(A)** Flow cytometry dot plots showing the effects of 24 h treatment with increasing concentrations of Sunitinib (top row) and Everolimus (bottom row) on cell death. Cells were stained with Hoechst 33342 to assess DNA content and Propidium Iodide (PI) to evaluate membrane integrity. The plots display the distribution of live, apoptotic, and necrotic cells after treatment with 20 µM, 40 µM, and 80 µM of each drug. **(B)** Bar graph quantifying the proportion of dead cells (sum of apoptotic and necrotic cells) after 24 h treatment with Sunitinib and Everolimus at the indicated concentrations. The percentage of dead cells is plotted against the concentration of the drug. Statistical analysis was performed using one-way ANOVA, with significance levels indicated as ***$p < 0.001$ compared to the control. **(C)** IC_{50} values of various inhibitors in iHemEC. The plot shows the IC_{50} values (µM) for different classes of inhibitors, including ACE inhibitors, Ang-Tie inhibitors, Notch pathway inhibitors, HIF1A inhibitors, ATR inhibitors, VEGFR inhibitors, and mTOR inhibitors, tested in iHemEC. Each point represents the mean IC_{50} value with error bars indicating the standard deviation. **(D)** KEGG pathway analysis of DEGs following Sunitinib treatment in iHemEC. Pathways are ranked by GeneRatio, with dot size indicating the number of associated genes and color representing the padj.
