# Supplementary material for: Everolimus and Sunitinib potentially work as therapeutic drugs for infantile hemangiomas
Source: Pediatr Res. 2025 Apr 5;98(6):2374–84. doi: 10.1038/s41390-025-04028-7 (PMC12811115; doi:10.1038/s41390-025-04028-7)
Supplement: Supplementary file 2 — Supplementary tables [file 41390_2025_4028_MOESM2_ESM.pdf]

Table S1 Screening of drugs with potential therapeutic capabilities for IH based on immortalized HemEC.

**Drugs**	**ID (Target Mol, Shanghai, China)**	**CAS Number**
Oltipraz	T0153	64224-21-1
HIF-1 inhibitor-4	T67767	333357-56-5
PX-478	T6961	685898-44-6
Sorafenib	T0093L	284461-73-0
Sunitinib	T0374L	557795-19-4
DAPT	T6202	208255-80-5
RO4929097	T6274	847925-91-1
Crenigacestat	T3633	1421438-81-4
Rapamycin	T1537	53123-88-9
Everolimus	T1784	159351-69-6
Rebastinib	T2640	1020172-07-9
BAY-826	T38930	1448316-08-2
Captopril	T1462	62571-86-2
Ramipril	T1645	87333-19-5
VE-821	T3032	1232410-49-9
Ceralasertib	T3338	1352226-88-0
Elimusertib	T7318	1876467-74-1

Table S2 Primary and secondary antibodies for western blot and immunofluorescence staining.

**Antibodis**	**Brand name**	**Product number**
VEGFR2	Cell signaling technology	9698S
CD31	Santa Cruze	sc-53411
GLUT-1	Maixin Biotech	MAB-0813
p-p53	Abmart	T55211
P53	Abmart	TA0879
Bax	Abmart	T40051
Bcl2 Rabbit mAb	Abmart	T40056
cMyc Rabbit mAb	Abmart	TA0358
p-AKT Rabbit mAb	Cell signaling technology	4060S
AKT Rabbit mAb	Cell signaling technology	4691S
p-mTOR Rabbit mAb	Cell signaling technology	5536S
mTOR Rabbit mAb	Cell signaling technology	2983S
p-PI3K Rabbit mAb	Cell signaling technology	4228S
PI3K Rabbit mAb	Cell signaling technology	4249S
p-NFkB Rabbit mAb	Cell signaling technology	3033S
NFkB Rabbit mAb	Cell signaling technology	8242S
TNFα Mouse mAb	Santa Cruze	sc-52746
IL17	Abmart	TD6127
β-Actin Rabbit mAb	Cell signaling technology	4970S
β-tubulin Rabbit mAb	Cell signaling technology	2146
GADPH Rabbit mAb	Cell signaling technology	5174S
Alexa Fluor 488 Anti-mouse IgG	Abcam	ab150119
Alexa Fluor 647 Anti-rabbit IgG	Abcam	ab150081
Anti-rabbit IgG, HRP-linked Antibody	Cell signaling technology	7074P2
Anti-mouse IgG, HRP-linked Antibody	Cell signaling technology	7076S
